# Management of Insane Asylums

**Published:** 1880-05

**Authors:** 


### MANAGEMENT OF INSANE ASYLUMS.

THE officers of the New York State Lunatic Asylum have been subjected to considerable criticism of late, by reason of accusations, which, to say the least, have been brought forward in a hasty and ill-advised manner. The alleged mismanagement was first made public through a sensational article in a New York daily, thus arousing the anxiety of all the friends of the insane.

Inasmuch as the statute provides a certain remedy for any such evils, when duly proven to exist, it seems hardly right to cast suspicion upon a number of intelligent physicians without first establishing the truth of every assertion made.

The attention of the Legislature was subsequently called to the management of the Asylum, but in view of the facts presented, the Committee on Public Health, in their report to the Senate, virtually acquitted the officers of the institution of all the charges made. The New York Neurological Society at once answered this report and, in consequence, agitated still more the subject of the government of the Asylum in question.

As one of the results of this discussion a bill has been proposed, which provides that the State Board of Charities may exercise such authority over the various asylums, as now belongs to them individually. While there may be advantages in a law of this kind, there are also obvious objections to outside interference with matters of local interest, and any such change should be made only after careful consideration, and with the approval of the great majority of medical men.

